# A Fluorometric Method of Measuring Carboxypeptidase Activities for Angiotensin II and Apelin-13

**DOI:** 10.1038/srep45473

**Published:** 2017-04-05

**Authors:** Pan Liu, Jan Wysocki, Peter Serfozo, Minghao Ye, Tomokazu Souma, Daniel Batlle, Jing Jin

**Affiliations:** 1 Division of Nephrology and Hypertension; The Center for Kidney Research and Therapeutics at the Feinberg Cardiovascular Research Institute, Northwestern University Feinberg School of Medicine, Chicago, IL, USA

## Abstract

Degradation of the biologically potent octapeptide angiotensin Ang II-(1-8) is mediated by the activities of several peptidases. The conversion of Ang II to the septapeptide Ang-(1-7) is of particular interest as the latter also confers organ protection. The conversion is catalyzed by angiotensin-converting enzyme 2 and other enzymes that selectively cleave the peptide bond between the proline and the phenylalanine at the carboxyl terminus of Ang II. The contribution of various enzyme activities that collectively lead to the formation of Ang-(1-7) from Ang II, in both normal conditions and in disease states, remains only partially understood. This is largely due to the lack of a reliable and sensitive method to detect these converting activities in complex samples, such as blood and tissues. Here, we report a fluorometric method to measure carboxypeptidase activities that cleave the proline-phenylalanine dipeptide bond in Ang II. This method is also suitable for measuring the conversion of apelin-13. The assay detects the release of phenylalanine amino acid in a reaction with the yeast enzyme of phenylalanine ammonia lyase (PAL). When used in cell and mouse organs, the assay can robustly measure endogenous Ang II and apelin-13-converting activities involved in the renin-angiotensin and the apelinergic systems, respectively.

The renin-angiotensin-aldosterone system (RAAS) regulates blood pressure and fluid balance and is the key target of many pharmacologic interventions in the treatment of cardiovascular and kidney diseases^1^,^2^,^3^. Clinical tests to evaluate RAAS were developed decades ago namely for plasma renin activity (PRA) and plasma ACE activity^4^,^5^,^6^, the latter also used in the diagnosis of sarcoidosis^7^,^8^,^9^.There is growing interest in the degradation of Ang II and the enzymes involved^10^,^11^,^12^. Assays for ACE2 and other Ang II-degrading enzymes employ fluorimetric and non-fluorimetric methods^13^,^14^,^15^. Methods for detecting Ang II and several other downstream peptides are also available although rarely used in the clinical setting. Among the degradation products of Ang II, the Ang-(1-7) peptide is of particular interest because of its cardiac and renoprotective actions^10^,^16^,^17^,^18^,^19^.

Proteolytic removal of the carboxyl terminal phenylalanine (Phe^8^/F) residue to form Ang-(1-7) is achieved by several peptidases that include angiotensin converting enzyme 2 (ACE2), prolyl carboxypeptidase (PrCP)^20^,^21^, and prolyl endopeptidase (PEP/PrEP)^12^,^20^,^22^. Of note, very little is known about the relative strength and actions of enzymes other than ACE2 that form Ang-(1-7) from Ang II (1-8)^23^. This limited information is due in part to the short half-life of Ang-(1-7) and other downstream metabolites, such as Ang-(1-5)^24^. Methods to quantitatively measure Ang-(1-7)-producing activity include the antibody-based Ang immunoassays, such as radioimmunoassay and ELISA^12^,^25^. However, antibody cross-reactivity with other angiotensin peptides is potentially confounding. Another method uses mass spectrometry-based detection of Ang-(1-7) formation following incubation of synthetic Ang II with tissue sections^26^ or tissue lysates^15^. All these methods involve time-consuming sample preparation that is susceptible to continuous degradation of Ang-(1-7) during the procedures and thus may increase experimental variability. Also, the Ang-(1-7) concentration in tissue samples is a moving target as its degradation by ACE and possibly other peptidases occurs rapidly^24^.

To circumvent these problems, we have developed an assay to evaluate conversion of Ang II to Ang-(1-7), as compared to that driven by recombinant mouse ACE2 as an exogenous control and the combined activities of endogenous Ang-(1-7) forming enzymes naturally expressed in organs. This method takes advantage of the fact that Ang II can only be converted to Ang-(1-7) by splitting phenylalanine (Phe) from the carboxyl end of Ang II. The phenylalanine-based assay described in this report does not capture the formation of peptides other than Ang-(1-7) resulting from Ang II (1-8) cleavage. It therefore provides a specific approach to study enzymes that convert Ang II to Ang-(1-7) when Ang II is used as the substrate. We note that a similar concept was used before^27^,^28^. However, the validity of the general approach pertinent to catalytic parameters was not investigated comprehensively or in any detail. Instead, we systematically studied the reactions using peptidase ACE2 as a benchmark model in both simple and complex systems. We now demonstrated the usefulness of the method, and established an optimized working protocol that greatly expands the general applications of the method. Because the amino acid phenylalanine is stable in any tissue lysis conditions, the method is also amenable for experiments designed to screen for new enzymes that degrade Ang II and form Ang-(1-7). This fluorescence-based assay is time-saving, quantitative and reliable to measure specific Ang II to Ang-(1-7) converting activity in complex biological samples. In addition, we tested this assay with another peptide substrate, apelin-13, which also plays a role in cardiovascular disease^29^. Since apelin-13 can also be degraded through proteolytic removal of the carboxyl terminal phenylalanine (Phe^13^/F) residue^30^, we reasoned that the proposed phenylalaninine assay should be able to also detect the cleavage of apelin-13 by ACE2.

## Results

### Phenylalanine assay with coupled fluorogenic reactions

**Figure 1:**
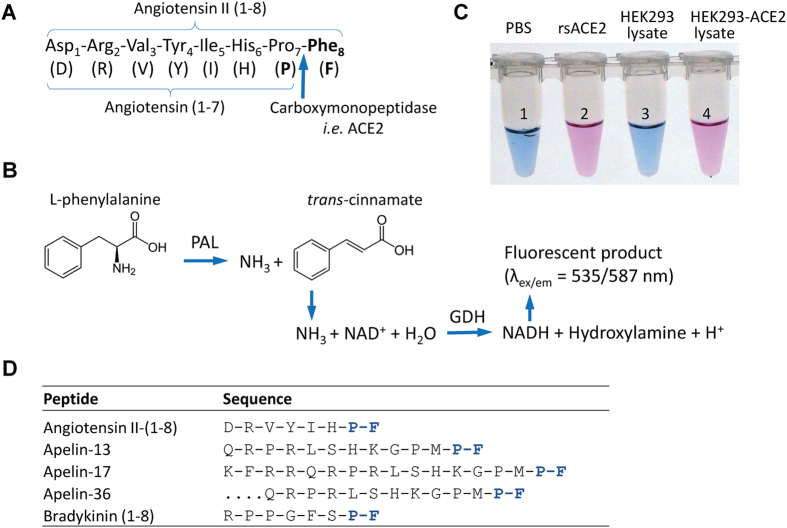
Schematics of the Ang II phenylalanine assay. (**A**) The assay measures the rate of Ang II cleavage at the peptide bond of proline_7_-phenylalanine_8_ by ACE2 or other equivalent carboxymonopeptidases (arrow). (**B**) Following the proteolytic cleavage, the amino group of phenylalanine becomes exposed to undergo deamidation by yeast enzyme phenylalanine ammonia lyase (PAL), also termed phenylalanine deaminase. Following a series of coupled reactions, a fluorogenic substrate is converted, and the rate of conversion that reflects the kinetic of the first reaction can be quantitatively measured. (**C**) The picture shows the color change as the result of the chain of the reaction initiated by ACE2 cleavage of synthetic Ang II peptide. Tubes 1 and 2 are from reactions in the absence and presence of recombinant soluble ACE2 (rsACE2: aa 1–740), respectively; tubes 3 and 4 are each from reactions with the addition of culture medium from cells transfected with either pcDNA3 empty vector or a soluble ACE2-expressing plasmid respectively. Positive ACE2 enzymatic activities in 2 and 4 convert fluorogenic substrate to bright pink color visible to naked eyes (with emission at 587 nm). (**D**) Several bioactive peptides share the same proline-phenylalanine (P-F) motif at their C-termini.

We first developed the assay using Ang II as the substrate to form Ang-(1-7). The new method involves a series of four reactions using synthetic Ang II as the starting substrate. The Ang-II-converting activity is measured as the production rate of L-phenylalanine (Phe) following proteolytic cleavage of the Pro^7^-Phe^8^ peptide bond at the carboxyl terminus (the schematics of the reaction is in Fig. 1). In the first reaction, Ang II is allowed to react with purified converting enzymes (such as ACE2) or with tissue lysate, in which Ang II to Ang-(1-7) conversion releases the carboxyl terminal Phe^8^ (Fig. 1A). In a coupled reaction, the exposed primary amine (-NH_2_) group of phenylalanine amino acid is hydrolyzed by phenylalanine ammonia-lyase (PAL) of *Rhodotorula glutinis* and ultimately turns on a fluorescent probe (Fig. 1B). For illustrative purposes, the color conversion - visible to naked eyes - following the reactions is shown in Fig. 1C. Typically, the reaction sets are operated in a 96-well format and either dynamic or endpoint reading of fluorescence excitation intensity is automated (details in Methods).

Since the new assay detects the cleavage of a carboxyl phenylalanine from polypeptides, it is worth noting that a few other biologically active peptides also share the proline-phenylalanine (P-F) dipeptide motif signature (Fig. 1D). Among them is apelin-13, a peptide also cleaved by ACE2^30^. ACE2 can cleave the peptide bond preceding Phe^77^ 31 causing inactivation of apelin^32^. A potent stimulator of cardiac contractility, apelin exists in a number of active polypeptide forms: apelin-13 (aa 65–77), apelin-17 (aa 61-77) and apelin-36 (aa 42–77), all carry the dipeptide motif at their carboxy termini and are expected to react with ACE2. We therefore asked whether the new phenylalanine assay can also be adapted to measuring carboxypeptidase activities that degrades apelin-13 (see below).

### The robustness and dynamic performance of the assay toward Ang II and apelin-13 substrates

**Figure 2:**
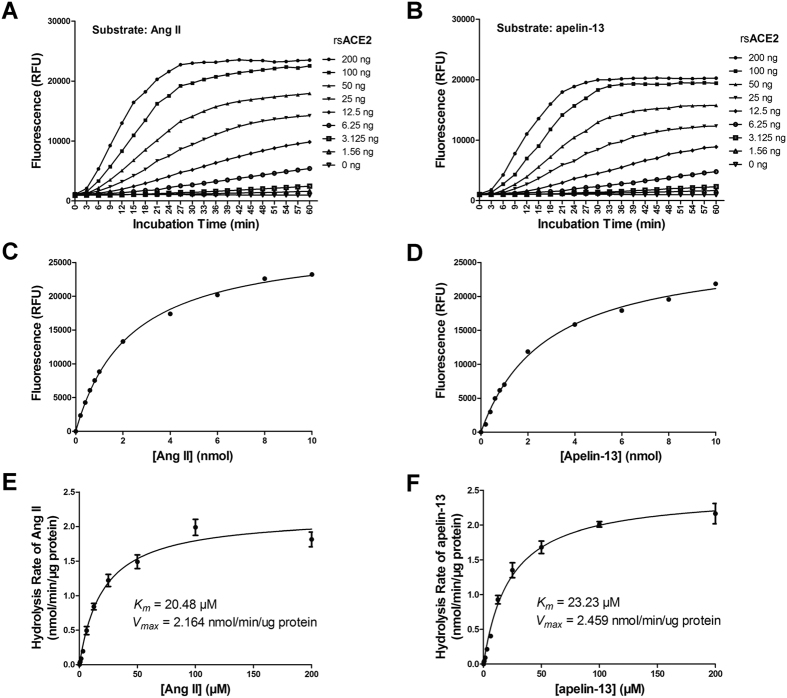
The *in vitro* phenylalanine assay for ACE2 activity. (**A,B**) Kinetics of enzymatic activities toward Ang II (**A**) and apelin-13 (**B**) substrates at 10 nmol per reaction with different concentrations of rsACE2. (**C,D**) Dose response curves of rsACE2 at situated concentration of 1 μg against Ang II (**C**) and apelin-13 (**D**) of indicated concentrations. (**E**,**F**) Kinetics of ACE2 activity measured by phenylalanine assay. The *K*_*m*_ and *V*_*max*_ for Ang II (**E**) and apelin-13 (**F**) were calculated based on Michaelis-Menten plot. Values are expressed as the mean ± S.D. from triplicate incubations.

ACE2, an enzyme that cleaves both Ang II and apelin-13, was used in these studies to validate the phenylalanine assay with each of these two substrates. We have previously made recombinant soluble ACE2 (rsACE2 of murine: aa 1-740 of the extracellular catalytic segment) and tested its activity using Mca-APK(Dnp)^33^. We sought to determine the dynamic performance of the assay in dose-response studies (Fig. 2). Eight reactions with two-fold serially diluted rsACE2 were monitored for over 60 minutes in presence of phenylalanine assay regents (Fig. 2A–B). With the same amount of substrate in each reaction, both Ang II and apelin-13 gave similar results (A compared to B), showing that the reaction conditions had allowed complete dose separation in each series. The results also showed that relative fluorescence intensity reached a plateau within 30 min in the highest ACE2 groups (Fig. 2A–B). Therefore, we used 30 min as the optimized first-step reaction time for the following two-steps phenylalanine assays (details in Methods).

To evaluate the suitability of the phenylalanine assay for quantitative analysis, we performed analysis of the Ang II and apelin-13 converting activity using rsACE2. First, we incubated an excess quantity of ACE2 in reactions with variable amount of substrate for 30 min followed by phenylalanine reaction. The substrate-fluorescence dependent curve was derived using non-linear regression (Fig. 2C,D). Then, we performed phenylalanine assay to calculate the kinetic parameters for rsACE2 by measuring the catalysis rate against Ang II and apelin-13, respectively. The *K*_*m*_ value of rsACE2 for Ang II was 20.48 μM and that for apelin-13 was 23.23 μM. The *V*_*max*_ value for Ang II was 2.164 nmol/min/ug protein and for apelin-13 was 2.459 nmol/min/ug protein (Fig. 2C,D). Of note, rsACE2 showed very similar catalytic efficiencies toward Ang II and apelin-13 substrates.

**Figure 3:**
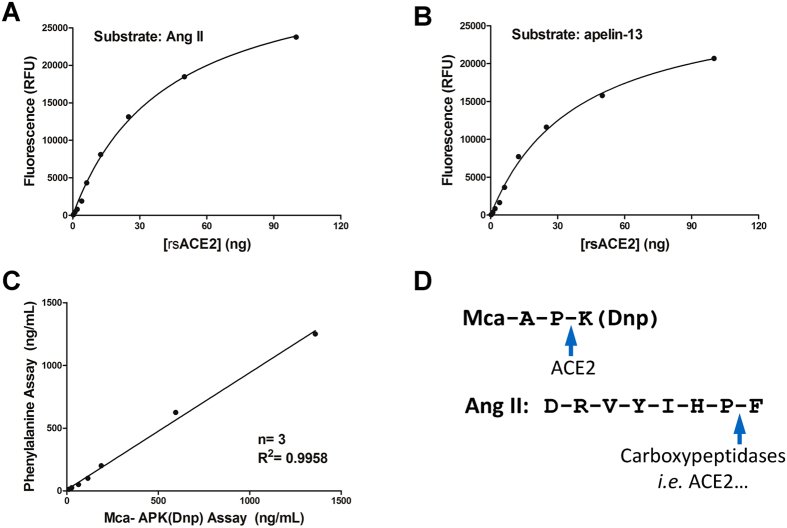
(**A,B**) Dose-response curves for rsACE2 by the phenylalanine assay. A shows reaction with 10 nmol Ang II peptide; and B with 10 nmol apelin-13 peptide. (**C**) The ACE2 activities as separately measured by the Ang II phenylalanine assay and the Mca-APK(Dnp) assay strongly correlate with each other. (**F**) Distinct amino acid sequences of Mca-APK(Dnp) and Ang II. The arrows point to the peptide bonds cleaved by ACE2, and in the case of Ang II also by other carboxymonopeptidases.

For the reason that one of the intended applications of the assay are to measure converting activities in biological fluids or tissues, which may possess a lower level of enzymatic activities, we tested the analytical performance of the assay by measuring the activity from various concentrations of rsACE2 using Ang II or apelin-13 as substrate and plotted standard curves. Because the system produces virtually no background signals, as low as 0.25 ng of the enzyme could still be sensitively detected (Fig. 3A and B). Good linear ranges for ACE2 activity were obtained from 0.25 to 4 ng on a non-logerithmic scale with *R*^2^ = 0.9955 for Ang II and *R*^2^ = 0.9968 for apelin-13, and from 4 ng to 100 ng on a logarithmic scale with *R*^2^ = 0.9946 for Ang II and *R*^2^ = 0.9971 for apelin-13 (supplementary Figure S2A–D).

We next evaluated the intra-assay and inter-assay precision and reproducibility. Samples that contain different rsACE2 amounts were measured in reactions with Ang II, and the results were subsequently calculated from the standard curves obtained above. Supplementary Table S1 shows the intra-assay coefficient of variation (CV) ranged from 2.12% to 7.4% and the inter-assay CV ranged from 6.23% to 10.1%. These low CV values indicate high reliability of the assay.

We also compared the ACE2 activity results obtained from this phenylalanine assay with the existing Mca-APK(Dnp) method. rsACE2 in a serial dilution series was added to reactions with either Mca-APK(Dnp) or Ang II peptides, and measured activities of ACE2 were directly compared. Both methods measured ACE2 activities that not only correlated with the input concentrations of the enzyme, but also highly correlated to each other (*R*^2^ of 0.9958, Fig. 3C). Here we should emphasize that the two substrates are of rather distinct sequences (Fig. 3D), and Mca-APK(Dnp), which sequence was originally designated for caspase 1, as a surrogate can be cross-recognized by ACE2^34^. Therefore, the ACE2 specific inhibitor MLN-4760 are used in conjunction with MCA-APK(Dnp) to measure activity exclusively attributed to ACE2^33^. Instead, the phenylalanine assay, measures either ACE2-dependent or combined total converting activities against Ang II when used in presence or absence of this ACE2 inhibitor, respectively.

### The phenylalanine assay used on a cell system

**Figure 4:**
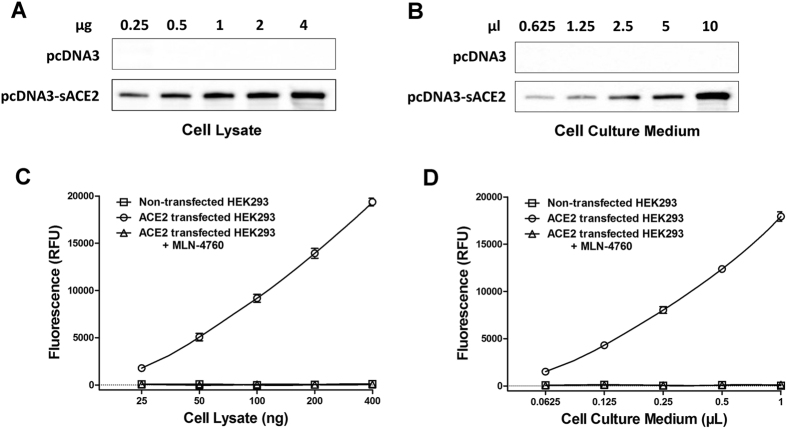
ACE2 expressed by HEK293 cells can be detected by the Ang II phenylalanine assay. HEK293 cells were transfected with either vector alone (pcDNA3) or pcDNA3-ACE2-Fc. Cell lysate and culture medium were separately collected and were subsequently subjected to western blot (**A** and **B**) and the phenylalanine assay using Ang II substrate (**C** and **D**). Full-length blots/gels are presented in Supplementary Figure S3. ACE2 activities were detected and these activities were completely inhibited by MLN-4760. The results in **C** and **D** are expressed as the means ± SD of three independent experiments.

We next set out to test whether the new assay can faithfully measure ACE2 activity levels in a cell system. We adapted a HEK293 cell transfection system to express Fc tagged soluble ACE2, like in the previous examples shown in Fig. 1C. The expression of sACE2-Fc from pcDNA3-rsACE2 transfected cells was confirmed by western blot (Fig. 4A,B), and its activity could be detected in cell lysates and in the culture medium (Fig. 4C,D). These activities were completely abolished in the presence of ACE2 inhibitor MLN-4760 in the reaction. Also as expected, the control cells transfected with only empty plasmid produced no activity.

### Detection of injected rsACE2 activities in blood using the Ang II phenylalanine assay

**Figure 5:**
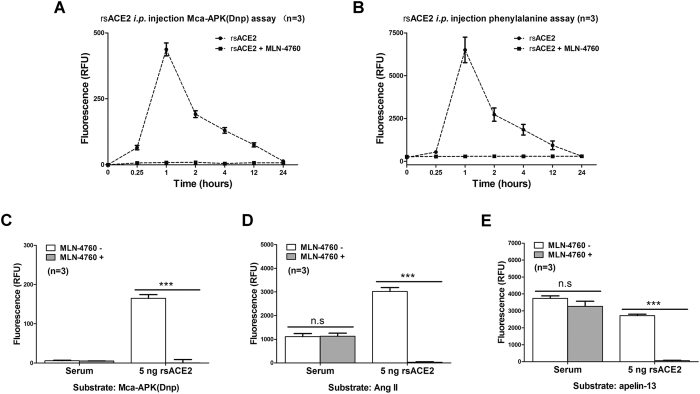
The phenylalanine assay detects carboxypeptidase activities against Ang II and apelin-13 in mouse serum. (**A,B**) Following a single intraperitoneal injection (*i.p.*) of 50 μg of rsACE2, tail blood was collected at different time points. ACE2 activity from 0.5 μL serum was measured by either Mca-APK(Dnp) (**A**) or Ang II phenylalanine assay (**B**) with or without MLN-4760 inhibition of ACE2. (**C**) Mca-APK(Dnp) measured endogenous ACE2 activity with or without MLN-4760 in untreated mice serum samples. (**D,E**) Phenylalanine assay with 5 μL serum from untreated mice using Ang II (**D**) or apelin-13 (**E**) as substrate. 5 ng rsACE2 was used as positive control in C through E. The results are expressed as the means ± SD of three independent experiments. (***p < 0.001, n.s: not significant, *t* test).

We sought to use the new assay, in a comparison with Mca-APK(Dnp), to measure ACE2 activities in serum samples. Following a single *i.p.* injection of 50 μg rsACE2, increase of ACE2 activities could be detected as early as 15 minutes (Fig. 5A,B). The levels peaked at about 1 hour and gradually dissipated over a 24-hour period. Overall, the two methods showed virtually identical serum profiles of ACE2 activity (n = 3), indicating that the new Ang II phenylalanine method can sensitively measure ACE2 activity in complex serum samples. ACE2-specific inhibitor MLN-4760 was used as negative control.

### Endogenous carboxypeptidase activities for Ang II and apelin-13 in mouse serum

Full length ACE2 is a transmembrane protein and only minimally present in serum through protease-mediated shedding^35^. We separately performed Mca-APK(Dnp) and phenylalanine tests of mouse serum. Recombinant ACE2 was used as a benchmark control in order to cross-reference between the two assays. As previously reported by us and others^33^,^36^,^37^, there is minimal serum ACE2 activity measured for Mca-APK(Dnp) (Fig. 5C). By contrast, by using a large volume of mouse serum (5 μL) we detected converting peptidase activities comparable to that of 5 ng rsACE2 as measured by the phenylalanine assay using Ang II and apelin-13 peptides (Fig. 5D and E respectively). These results suggest that Ang-(1-7) formation from Ang II in serum is to a large extent ACE2-independent, although this converting activity is relative low and might be undetectable as reported by others using other methods that used a small amount of serum^15^. Carboxypeptidases reacting to Ang II and apelin-13 other than ACE2 contribute therefore to total phenylalanine intensity (particularly at the tissue level) (see below).

### Mouse organ activities measured by phenylalanine assay

Next, we sought to measure Ang II-converting activities in complex tissue lysates. First, we conducted control experiments to determine the background levels of the assay from two contributing sources: The naturally existing phenylalanine in tissues and phenylalanine resulted from non-Ang II substrates such as abundant tissue proteins. Using mouse kidney lysate, even without Ang II substrate, there was a background level of phenylalanine reading likely from endogenous phenylalanine. As the reaction proceeded with Ang II, the reading of phenylalanine levels increases steadily over time (supplementary Fig. S1A). This is in stark contrast to the reaction that maintained a constant intensity of phenylalanine signal when Ang II was absent, suggesting that reaction signals were derived from Ang II-conversion, instead from any protein substrates in tissue lysate. Endogenous phenylalanine levels tend to vary (up to 2 mg/dL in blood) depending on diet. We next performed desalting of the lysate to remove endogenous phenylalanine (details in Methods) and then subjected the resulting tissue lysates to phenylalanine assay (no Ang II added to the reactions). As expected, desalting reduced background intensities in a majority of mouse tissues tested, most notably in serum (supplementary Fig. S1B). Therefore, we adapted this desalting step in tissue preparation in subsequent experiments. The assay can equally be performed without the step but then it requires subtraction from baseline phenylalanine activity.

**Figure 6:**
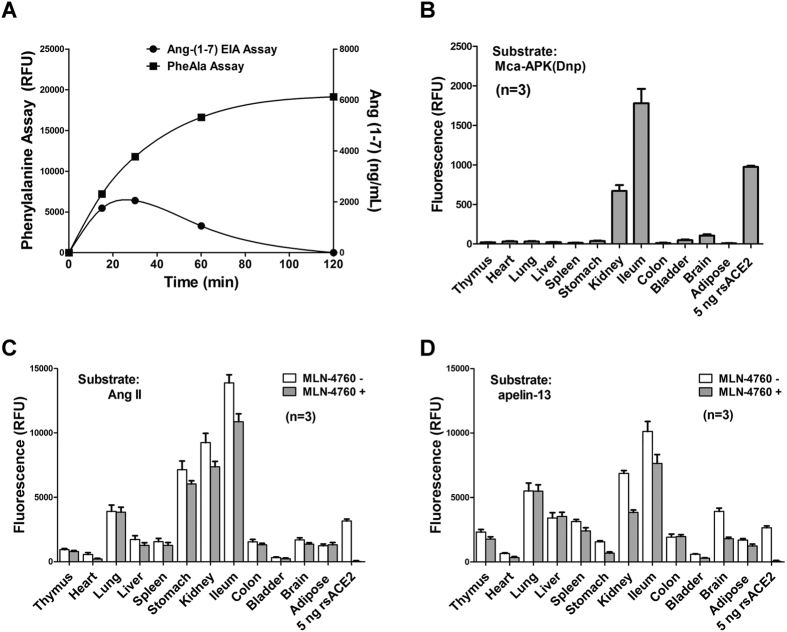
Tissue distribution of carboxypeptidase activities for Ang II and apelin in mice. (**A**) Concurrent formations with distinct dynamic trends of phenylalanine and Ang-(1-7) following the incubation of Ang II with mouse kidney lysate. (**B**) ACE2 activity in mice tissue lysates (10 μg) was measured by the Mca-APK-Dnp method. Carboxypeptidase activities for Ang II (**C**) and apelin-13 (**D**) in mouse tissues were measured by the phenylalanine assay. The results are expressed as the means ± SD of three independent experiments.

In addition, we measured the kinetics of Ang-(1-7) formation in reactions of Ang II mixed with kidney lysates. Following the initial accumulation of Ang-(1-7) that peaked at 30 min, its total levels gradually subsided that likely reflect further degradation of newly formed Ang-(1-7). By contrast, phenylalanine was stably produced throughout the course of the 120 min reaction (Fig. 6A). Evidently, the net Ang-(1-7) levels were influenced by the rates of its formation and degradation, and the commonly used EIA method may not truly represent Ang-(1-7) formation activity. By contrast, this activity can be better captured by the phenylalanine assay attributed to the stability of phenylalanine amino acid.

We conducted the assay on 12 mouse organs. For comparison, the Mca-APK(Dnp) assays were performed with and without ACE2 inhibition and ACE2 activity levels were estimated as the difference between the two measurements. Consistent with published results^38^, ACE2 activities were prominently present in the ileum followed by the kidney (Fig. 6B). When the same tissue lysates were subjected to phenylalanine reactions using either Ang II or apelin-13 as substrates (Fig. 6C and D respectively), the ileum and kidney remain the highest in activity. In addition, some other organs including lung and stomach also display high carboxypeptidase activities. It is interesting to note that MLN-4760 inhibition of ACE2 reduced phenylalanine activity to a variable extent, most noticeably in ileum, kidney and heart, in which ACE2 has been shown to play an essentially role in Ang II catabolism^15^,^38^,^39^.

## Discussion

We have devised a fluorometric assay to measure carboxypeptidase-mediated degradation of Ang II to form Ang-(1-7). The method is sensitive in detecting activities from cell and organ lysates based on the detection of the hydrolysis of the carboxyl terminal phenylalanine of the octapeptide Ang II. Similarly, the cleavage of the carboxyl terminal phenylalanine of apelin-13 can also be easily and accurately detected by this assay. By using the carboxy(mono)-peptidase ACE2 as a control paradigm, we optimized the reaction conditions to achieve linear performances over a wide dynamic range.

In RAAS, Ang II is converted by several peptidases include ACE2 to produce Ang-(1-7), which, through acting on its own receptor Mas^40^, counterbalances the effects triggered by Ang II^41^. This catalytic property has evoked interest in these enzymes, especially ACE2, as a possible therapeutic target for cardiovascular and renal diseases treatment^10^,^33^,^42^,^43^. ACE2 is also the only known enzyme to catalyze apelin-13, which is the dominant peptide in apelin family^30^. Currently, the ACE2 activity is commonly measured by using fluorometric substrates Mca-YVADAPK(Dnp) and Mca-APK(Dnp). Here, by using Mca-APK(Dnp) assay as a benchmark, we demonstrated that phenylalanine assay produced remarkably consistent results for measuring ACE2 activities (Fig. 3E). The phenylalanine assay comprehensively measures the Ang-(1-7) forming activity, which may complement the ACE2 activity assay to evaluate Ang II metabolism.

ELISA-based methods that measure the input of Ang II or the production of Ang-(1-7), or both, are available^12^,^25^. However, antibody-based assays are intrinsically finicky and the input substrate peptide and the tissue lysate amount have to be carefully optimized to achieve linearity. Even with the best setting, the dynamic range is less than one order of magnitude, as opposed to the three orders of magnitude range achieved by the Ang II phenylalanine assay (Figs 2 and 3). The clinical importance of the RAAS in a multitude of diseases underscores the need for techniques to measure enzymatic activities in biological samples, such as blood and tissues. The new method could have broad applications for analyzing biological and clinical samples. For instance, when used with pure enzymes including those known to have Ang II converting activities, such as prolylcarboxypeptidase (PrCP)^44^,^45^, prolylendopeptidase (PEP/PrEP)^46^, alongside ACE2, it may provide meaningful comparison of kinetic parameters as well as tissue specific distribution and activity of these carboxypeptidases. Furthermore, since we demonstrated that the assay is also suited for analyzing serum and tissue lysates, potential applications of the technique to clinical samples are possible. The sample volume required for the test is diminutive which makes it feasible for both research and clinical use. Of particular importance, the new test may shed light on regulations of Ang II degradation in health and disease states.

It is anticipated that organ lysates contain complex peptidase activities, including those contributing to continuing degradation of newly formed angiotensin peptides. Previous studies that measured Ang-(1-7) formation applied protease inhibitors such as PMSF and bestatin to suppress undesired protease activities^15^,^47^. However, the broad-specificity of these inhibitors may also compromise the effort on finding physiologically relevant converting enzymes that might be sensitive to inhibition. In contrast, the phenylalanine approach measures the levels of stable amino acid of phenylalanine, and therefore was used in the absence of general protease inhibitors. Still, we should note the caveats of the new assay. The phenylalanine results likely reflect the combined enzyme activities attributing to phenylalanine formation, including but not limited to Ang II to Ang-(1-7) conversion. For instance, concurrent proteolytic cleavage of Ang II peptide by additional enzymes might happen prior to the release of phenylalanine. Therefore, the new method, when used in conjunction with a selected combinations of specific peptidase inhibitors, may be pursued in the future towards better delineating *in vivo* Ang II to Ang-(1-7)-converting enzymes.

The new method can also be used to measure activities against apelin-13, which share the carboxyl terminal proline-phenylalanine motif with Ang II^30^. Other bioactive peptides including bradykinin can be catalyzed by peptidases through removing of the carboxyl phenylalanine (Fig. 1D). For the same matter, this assay should also work for them, although we have not experimentally tested that yet. Admittedly, the new phenylalanine assay also has its limitations when used on complex lysates because endogenous enzymes that would otherwise be spatially inaccessible by extracellular angiotensin peptides will react false positively in the *in vitro* assay. Nevertheless, based on our initial tests of the new phenylalanine assay with both simple and complex activities, this procedure is sensitive and robust. Applying the assay to a range of disease models such as hypertension and diabetic nephropathy can potentially yield important insights on disease mechanisms.

## Methods

### Materials

Ang II and apelin-13 peptides were purchased from Anaspec (San Jose, CA, USA). Phenylalanine detection kit was purchased from Sigma-Aldrich (St. Louis, MO, USA). ACE2 substrate Mca-APK(Dnp) was purchased from Enzo Life Sciences (Farmingdale, NY, USA). Anti-ACE2 antibody was from Abcam (Cambridge, MA, USA). ACE2 inhibitor MLN-4760 was a gift from Millennium Pharmaceuticals (Cambridge, MA, USA). The recombinant extracellular domain of mouse ACE2 (termed rsACE2) fused with 10 X His tag was generated by our lab and produced on large-scale by Invitrogen customer service (Carlsbad, CA, USA) as reported previously^33^.

### Phenylalanine assay for ACE2 activities against Ang II or apelin-13

To kinetically measure the rsACE2 activity by phenylalanine assay, rsACE2 was serial diluted with reaction buffer (20 mM Tris-HCl, pH = 7.4, 136 mM NaCl and 10 μM ZnCl_2_) starting from 0.2 mg/mL. 1 μL of the diluted enzyme was mixed with 1 μL Ang II (10 mM) or apelin-13 (10 mM) substrate, 1 μL enzyme mix and 1 μL developer from phenylalanine detection kit. The reactions were proceeded for 60 minutes at room temperature. Fluorescence intensity in a time series was measured at 535 nm excitation and 585 nm emission wavelength. All reactions were performed in triplicate.

To measure activities by endpoint fluorescence reading, peptidase reactions were proceeded at 37 °C in the presence of 0.5 μM Ang II or apelin-13. The reactions were stopped after 30 minutes by 80 °C heat inactivation for 5 minutes. The reaction mixtures were then directly added to phenylalanine detection buffer contain 1 μL enzyme mix and 1 μL developer in 96-well plates at 37 °C for another 20 minutes before fluorescence readings were measured. We note that removal of all protein contents before adding kit components as suggested by manufacturer’s instructions is not necessary, as our results in supplementary Figure S1A showed that protein contents neither increase background levels nor impede reaction. The reaction buffer for rsACE2, HEK293 cells and rsACE2-infused serum samples contains 20 mM Tris-HCl, pH = 7.4, 136 mM NaCl and 10 μM ZnCl_2_. For measuring endogenous activities in serum and tissue samples, before adding Ang II or apelin-13 substrate, the lysates were first desalted using Zeba^TM^ spin columns (Thermo Fisher Scientific, San Jos, CA, USA) – a step to remove endogenous phenylalanine and therefore reduce background readings. In consideration of a large number of peptidases including metallopeptidases may need divalent ions for their activities, the reaction buffer for desalted sera and tissue lysates contains additional 10 μM ZnCl_2_, 2.5 mM CaCl_2_ and 2.5 mM MgCl_2_. The reactions with tissue lysate was allowed to proceed at 37 °C for 30 minutes followed by heat inactivation, and then the phenylalanine assay. In the reactions using kidney samples, 10 μg desalted kidney lysates were incubated with 0.5 μM Ang II in a 20 μL reaction at 37 °C. At different time points the reactions were stopped by heating and the phenylalanine was measured as described above.

To measure the kinetic constants (*K*_*m*_ and *V*_*max*_) of enzyme, 0.1 μg rsACE2 was incubated with various concentrations of Ang II or apelin-13 substrate (0.195–200 μM) for 2 min followed by heating inactivation and phenylalanine detection. The rate of increase in fluorescence intensity in the initial phase (0–2 min) was calculated and converted to nmol/min/μg protein according to the substrate-fluorescence intensity standard curves. The values of kinetic constants *K*_*m*_ and *V*_*max*_ were obtained by fitting the corresponding data to the Michaelis–Menten equation using Graphpad Prism 5 software.

### Intra- and inter-assays

To evaluate intra-assay precision, five replicate samples containing low concentration (10 μg/mL), medium concentration (20 μg/mL) or high concentration (40 μg/mL) of ACE2 were prepared and measured using Ang II as substrate by the procedure described above. To evaluate inter-assay precision, the same low, medium and high concentration of ACE2 samples were prepared as intra-assay, and the measurements were repeated daily for 5 days. Mean and standard deviation (SD) of the samples were calculated, and then the coefficient of variation (CV%) was calculated as 100 × SD/mean.

### Phenylalanine assay for cells transfected with pcDNA3-ACE2-Fc

DNA fragment that encodes the extracellular domain of mouse ACE2 (aa 1-740) was fused with human IgG1 Fc domain and ligated into the *Hind III* and *Xho I* sites of pcDNA3 vector (Invitrogen). Human embryonic kidney cells (HEK293) were maintained in Dulbecco’s Modified Eagle Medium (DMEM) supplemented with 10% FBS and transfected with the pCDNA3-rsACE2 plasmid using lipofectamine-2000 (Invitrogen). 48 hours after transfection, culture media and the cell lysates were collected separately. The expression and enzymatic activities of rsACE2 were measured by western blot using anti-human IgG antibody (Thermo Fisher Scientific) and Ang II phenylalanine assay respectively. Reactions in the presence of ACE2 inhibitor MLN-4760 were negative controls.

### ACE2 activity against artificial Mca-APK(Dnp) substrate

ACE2 activity was measured by using Mca-APK(Dnp) as substrate in 96-well black microtiter plates as previously described^48^. The samples, either as rsACE2, cell lysate, cell culture medium, mouse serum or mouse tissue lysate, were added into wells containing reaction buffer (50 mM 4-morpholineethanesulfonic acid, pH = 6.5, 300 mM NaCl, 10 μM ZnCl_2_ and 0.01% Triton X-100,) with 20 μM of Ma-APK(Dnp). The total reaction volume was 100 μL and all experiments were performed in triplicate at room temperature for 30 minutes. Negative control reactions were proceeded in the presence of 10 nM MLN-4760, an ACE2 inhibitor. Activities were measured as fluorescence intensity at 320 nm excitation and 420 nm emission wavelength using an FLX800 microplate fluorescence reader (BIOTEK Instruments Inc., Winooski, VT, USA).

### Measurement of Ang-(1-7) levels by EIA

To measure the dynamic formation of Ang-(1-7) from an *in vitro* reaction with kidney lysate, 20 μL reactions containing 0.5 μM Ang II and 10 μg lysates were incubated at 37 °C. Reactions were stopped at different time points by diluting 1000 times with ice-cold methanol. After further diluting the sample in EIA buffer (1/50), the quantity of Ang-(1-7) was determined using an EIA kit (Peninsula Laboratories LLC, San Carlos, CA, USA), as per manufacturer’s instructions.

### Preparation of mouse tissues

The study of animals was approved by the Northwestern University Animal Care and Use Committee (Protocol #IS00000429), and all experiments were performed in accordance with relevant guidelines and regulations. Eight-week-old female 129/sv mice were purchased from Taconic Farms (Germantown, NY, USA). For the blood activity study, mice received intraperitoneal injection of 50 μg rsACE2 were subjected to tail-bleeding to collect blood samples in a time-series. Blood was left to clot at room temperature for 15 minutes, and was then centrifuged at 3000 × g for 10 minutes to collect serum. For rsACE2 injection experiment, 0.5 μL of serum was used in each reaction. For measuring endogenous peptidase activities, which tend to have lower activities, a larger volume of 5 μL was used in each reaction. In tissue sample preparation, we first removed blood through systemic perfusion with cold PBS. Tissues from these mice were homogenized in cold NP-40 lysis buffer (50 mM Tris, pH = 7.4; 150 mM NaCl and 1% NP-40). Clear lysates were obtained following centrifugation at 20,000 × g for 15 minutes at 4°C. After desalting using Zeba^TM^ column (Thermo-Fisher) to remove endogenous phenylalanine, total protein concentration was determined using Bio-Rad protein assay regent (Bio-Rad), and 10 μg of each sample was used in the final Mca-APK(Dnp) or phenylalanine assay as described above.

## Additional Information

**How to cite this article**: Liu, P. *et al*. A Fluorometric Method of Measuring Carboxypeptidase Activities for Angiotensin II and Apelin-13. *Sci. Rep.* **7**, 45473; doi: 10.1038/srep45473 (2017).

**Publisher's note:** Springer Nature remains neutral with regard to jurisdictional claims in published maps and institutional affiliations.

## Supplementary Material

Supplementary Figures and Tables
