# Supplementary material for: A Fluorometric Method of Measuring Carboxypeptidase Activities for Angiotensin II and Apelin-13
Source: Sci Rep. 2017 Apr 5;7:45473. doi: 10.1038/srep45473 (PMC5381230; doi:10.1038/srep45473)
Supplement: Supplementary Figures and Tables [file srep45473-s1.pdf]

Supplementary Information

A Fluorometric Method of Measuring Carboxypeptidase Activities for Angiotensin II and Apelin-13

Pan Liu, Jan Wysocki, Peter Serfozo, Minghao Ye, Tomokazu Souma, Daniel Batlle and Jing Jin

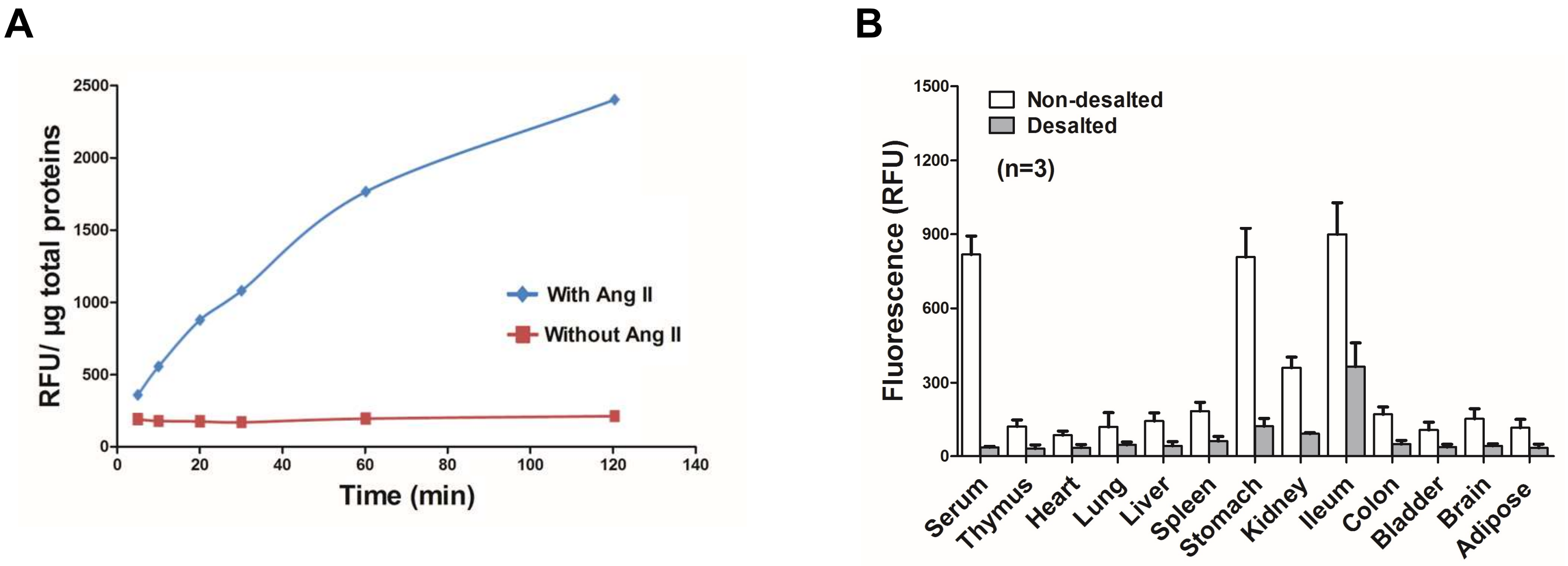

Supplementary Figure S1. Background controls for endogenous phenylalanine. A. Kinetics of phenylalanine reactions using kidney lysate in the presence (blue) or absence (red) of Ang II peptide as substrate. B. Background intensity in serum (1 µL) and in tissue homogenates (1 µg) with or without prior column desalting to remove endogenous phenylalanine.

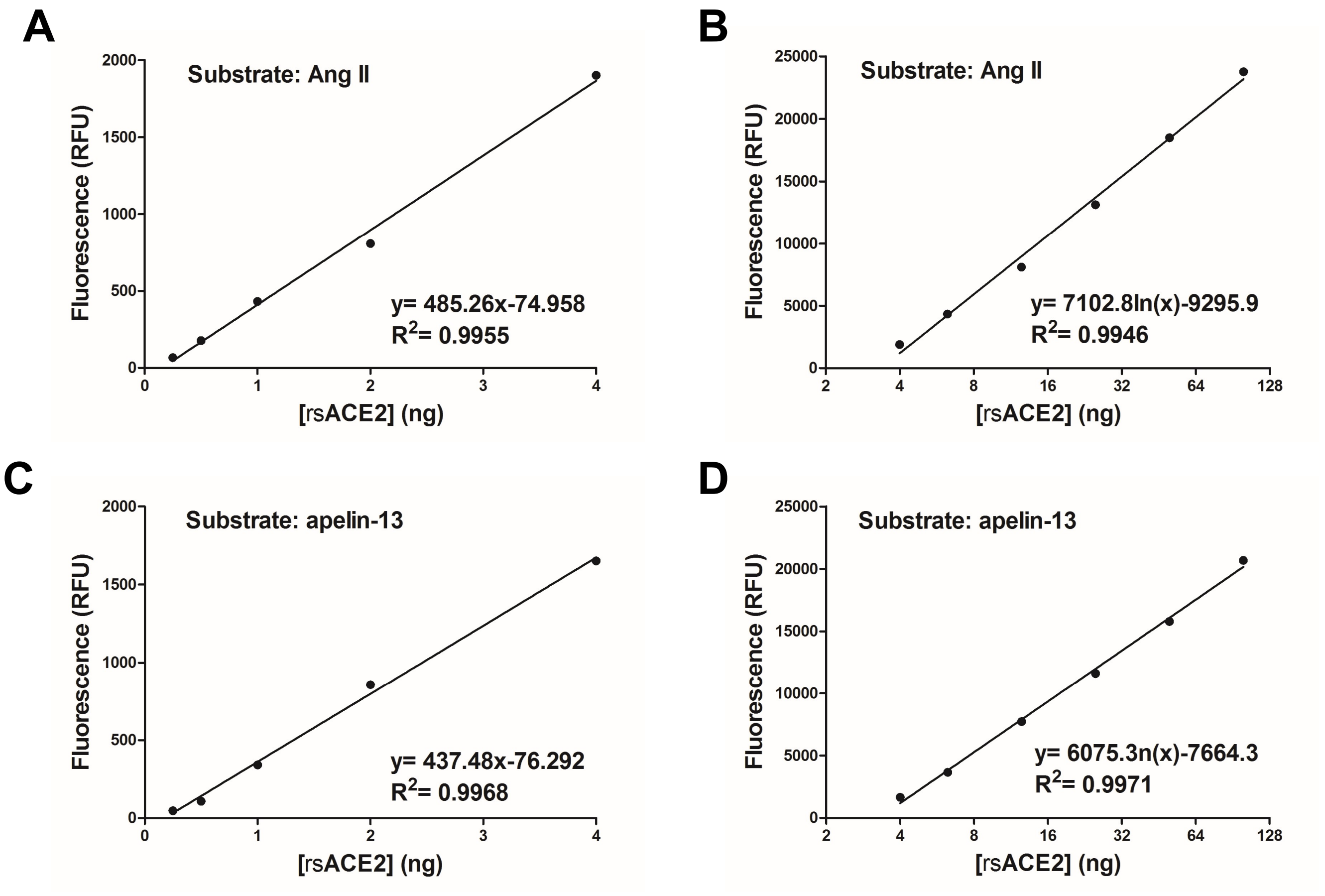

Supplementary Figure S2. Linear-fitting graphs derived from Figure 3A and 3B on either non-logarithmic *x*-axis scale for lower concentration of Ang II (A) and apelin-13 (C) (0.25 – 4 ng), or logarithmic *x*-axis scale for higher concentration of Ang II (B) and apelin-13 (D) (4 – 100 ng).

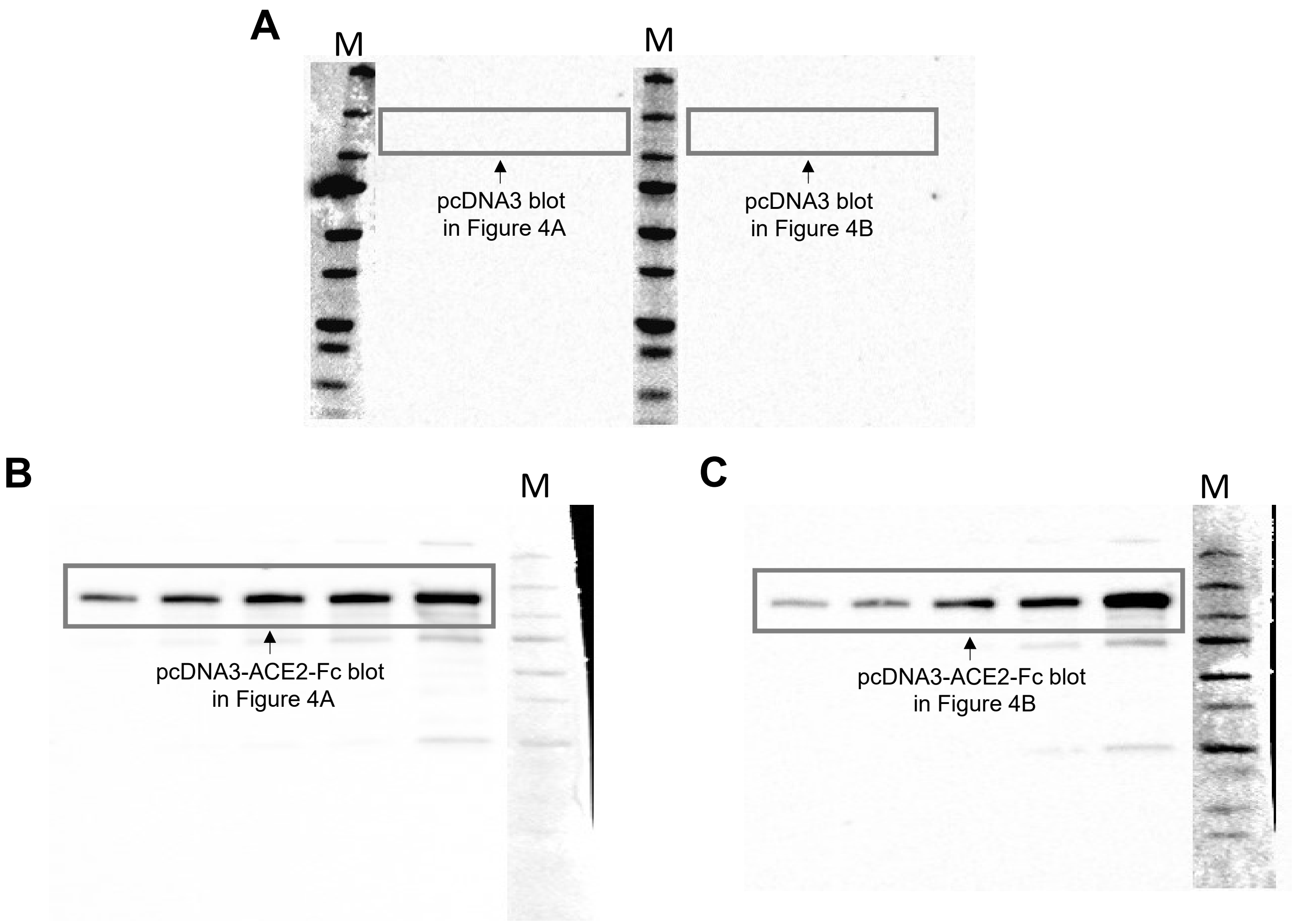

Supplementary Figure S3. Full-length images of Western blots and prestained marker standards (M). (A) Full-length blot corresponding to the pcDNA3 blot results shown in Figure 4A and 4B. **.** (B-C) Full-length blots corresponding to the pcDNA3-ACE2-Fc blot results shown in Figure 4A and 4B.

Supplementary Table S1. Intra- and inter-assay variation

rsACE2 concentration	Intra-assay precision (n=5)			Inter-assay precision (n=5)		
	Mean (ng/µL)	SD	CV (%)	Mean (ng/µL)	SD	CV (%)
High	44.05	3.26	7.40	43.76	3.70	8.46
Medium	20.86	0.94	4.51	18.79	1.17	6.23
Low	9.89	0.21	2.12	8.71	0.88	10.1

SD: Standard deviation, CV: Coefficient of variation.
